# Study on the ability of indoor plants to absorb and purify benzene pollution

**DOI:** 10.1038/s41598-024-63811-4

**Published:** 2024-06-07

**Authors:** Donghe Li, Han Wang, Qingyu Gao, Min Lu

**Affiliations:** 1 School of Architecture and Built Environment, Faculty of Science Engineering and Built Environment, Deakin University, Geelong, VIC 3220 Australia; 2 Landscape Architecture Research Center, Shandong Jianzhu Univerity, Jinan, 250101 Shandong China

**Keywords:** Benzene pollution, Indoor plants, Absorption and purification capacity, Purification rate, Purification amount, Restoration ecology, Urban ecology, Environmental impact, Sustainability

## Abstract

The ability of indoor plants to purify benzene pollution is the basic basis for the selection of plants for ecological remediation of indoor benzene pollution. In this study, the purification rate and the purification amount per unit leaf area of 13 test plants at three benzene concentrations were determined by indoor fumigation experiments, and the benzene absorption and purification abilityability of indoor plants were comprehensively evaluated. The results showed that (1) there was a significant correlation between benzene concentration and purification rate and purification amount per unit leaf area. (2) At the three concentrations, *Spathiphyllum floribundum* showed the highest purification rate and *Sansevieria trifasciata* var. *laurentii* showed the highest purification per unit leaf area. (3) The combined results showed that *Sansevieria trifasciata* var. *laurentii*, *Spathiphyllum floribundum* and *Aloe arborescens* were the strongest absorbers and purifiers, while *Podocarpus nagi* and *Anthurium andraeanum ‘Pink champin’* had the weakest absorption and purification capacity. The results of this study provide a theoretical basis and reference for the selection of plants with strong capacities to adsorb and purify benzene pollution in indoor air.

## Introduction

With the rapid development of society and economy, the problem of indoor environmental pollution is becoming increasingly serious^1^. Humanity has entered the "third pollution period", characterised by chemical pollution of indoor environments^2^. Currently, indoor levels of chemical pollution tend to be higher than outdoor levels^3^. Studies have shown that the average concentration of pollutants indoors is 5 to 10 times higher than outdoors, and that the indoor environment of newly renovated or refurbished buildings can contain levels of certain pollutants that are almost 100 times higher than outdoors^4,5^. People spend more than 80 per cent of their day indoors^6^, and indoor air quality is closely related to people's health^7^. Since the dissemination of COVID-19, improving indoor environmental quality has become a pressing global issue and a top priority for the scientific community today^8^.

A wide range of indoor chemical pollution problems caused by benzene and benzene systems have received increasing attention and concern^9,10^. Benzene, as one of the major pollutants in indoor chemical pollution volatile organic compounds (VOCs), is not only highly carcinogenic and teratogenic^11,12^, but also persistent and difficult to degrade^9^, which poses a serious threat to people's lives and health^13^. How to permanently, safely and effectively clean and control indoor chemical pollution has become an important issue to be resolved^3^.

Indoor plants not only have important carbon absorption and oxygen release functions^14^, but also have strong purification and remediation abilities for indoor chemical pollutants, especially with safe, stable, and sustained purification effects^15,16^. Studies have shown that ecological remediation of air pollution by plants relies mainly on the combined action of the plant itself, and plant root microorganisms^16^. It has been demonstrated that plants absorb indoor chemical pollutants into their bodies through stomata on their leaves and lenticels on their branches. These pollutants are then neutralized into non-toxic substances by plants through redox processes (i.e. degradation), either excreted through the root system or accumulated and stored in organs^17,18^. The process of adsorption, accumulation, decomposition and transformation of air pollutants by plants has been demonstrated to effectively achieve the absorption and purification of indoor chemical pollutants^19–21^. Therefore, the ecological remediation technology of indoor plants has become an effective and important means of managing indoor chemical pollution^22–24^.

The purification rate and purification amount per unit area of indoor plants for benzene pollution can effectively reflect the purification capacity of indoor plants for benzene pollution^18,25, 26^, and the purification capacity of indoor plants for benzene pollution is the basic basis for the selection of plants to purify indoor benzene pollution^27,28^. At present, relevant scholars have conducted many relevant studies on the purification ability and resistance of indoor plants to benzene and other indoor chemical pollution^12,29–31^. However, previous studies have mainly focused on determining the single purification rate^32–34^ or purification amount of indoor plants for benzene and other pollutants^35–39^, and few studies have been reported to comprehensively evaluate the purifying ability of multiple indoor plants against benzene pollution in terms of both plant purification rate and purification amount per unit area.

Therefore, 13 common suitable indoor plants in northern China were selected as materials in this study and exposed to three benzene concentration gradients (25 mg-m^–3^, 50 mg-m^–3^ and 100 mg-m^–3^) in an airtight fumigation test. The purification capacity of indoor plants for benzene pollution was comprehensively evaluated based on the changes in purification rate and purification amount per unit area of the experimental plants using the membership function method. The results of the study provide a theoretical basis and guidance for the selection of purifying plants for indoor benzene pollution.

## Materials and methods

### Experimental materials

Based on previous studies, 13 indoor plants with high resistance to indoor pollutants were selected as test material for the experiment, including *Chlorophytum comosum*, *Chlorophytum comosum* var. *variegatum*, *Aloe arborescens*, *Sansevieria trifasciata* var. *laurentii*, *Dieffenbachia picta*, *Spathiphyllum floribundum*, Epipremnum aureus, *Anthurium andraeanum 'Pink champin' Anthurium andraeanum*, *Podocarpus nagi*, *Kalanchee blossfeldiana*, *Begonia xaelator*, and *Calatha insignis*. According to the experimental process, the test plants were potted in the greenhouse provided by the Jinan Huamu Joint Development Company in batches. The plants were required to be grown in identical conditions, including their shape, pot material, size and soil dosage. Prior to the experiment, the plants designated for experimentation were acclimated to the laboratory environment for a period of two weeks.

### Experimental design

A randomized block design with threefold repetition was adopted for experiments, and an 80 cm × 80 cm × 80 cm cube glass airtight fumigation box was used to conduct airtight fumigation tests on 13 indoor plants. Previous studies by the research team revealed that physiological and biochemical indicators, such as POD, MDA, and chlorophyll Chl, exhibited significant alterations in indoor plants exposed to benzene concentrations of 25 mg·m^–3^, 50 mg·m^–3^, and 100 mg·m^–3^^2^. Therefore, to demonstrate the ability of indoor plants to purify benzene at high concentrations, a benzene gradient consisting of three concentrations of 25 mg·m^–3^, 50 mg·m^–3^, and 100 mg·m^–3^ was set up for fumigation in the experiments. Three replicates were established for each plant, with the plantless group as the blank control. To ensure the accuracy of the experiment and to avoid the effect of benzene absorption by the soil and pots, the soil in the pots and pot areas were wrapped in plastic film before the plants were placed in the sealed chamber for fumigation. After the plants were placed in the chamber, they were immediately sealed with tape to reduce the exchange of the gas inside the chamber with the outside environment. A small fan (220 V, 80W) is reserved in the fumigation box to speed up the volatilisation of pollutants; the room temperature is controlled at 23–25°C, and the humidity is 40–50%; wet and dry thermometers are placed to monitor changes in temperature and humidity in the chamber. After 24 h of fumigation, the concentration of benzene gas in the fumigation chamber was measured, and relevant experimental data and parameters were recorded. After the completion of fumigation treatment, the upper half of the tested plants was cut under the same benzene concentration, new shoots and leaves with the same growth duration and different orientations were cut to form a mixed leaf group, and indicators were measured.

### Indicator measurement

The concentration of benzene gas was measured using an Agilent 689 N gas chromatograph, and the leaf area of plants was measured using the paper weighing method^18^. The formula for calculating leaf area^29^ is as follows:

1 $$S={s}_{0}\times \frac{k}{{k}_{0}}$$

In the formula, S is the plant leaf area (m^2^), s_0_ is the total paper area (m^2^), k is the leaf type paper weight (g), and k_0_ is the total paper weight (g).

The formulas for calculating the purification rate (%) and purification amount per unit leaf area (mg·m^–2^·h^–1^) at different benzene concentrations^33^ are as follows:

2 $$A=\frac{{C}_{0}-{C}_{n}-C}{{C}_{0}}\times 100\%$$

where A is the benzene purification rate (%), C_0_ is the initial benzene concentration value in the fumigation chamber (mg·m^–3^), C_n_ represents the mass concentration of benzene in the n-hour in the fumigation chamber (mg·m^–3^), and C represents the concentration of pollutants in the plant-free control treatment (mg·m^–3^).

3 $${A}_{u}=\frac{\left({C}_{0}-{C}_{n}-C\right)\times V}{S}$$

In the formula, A_u_ is the purification amount per unit leaf area (mg·m^–2^·h^–1^), S is the plant leaf area (m^2^), and V is the volume of the fumigation box (m^3^).

### Statistical analysis

Excel software was used to compile the experimental data, SPSS 25.0 software was used to perform one-way ANOVA and two-way ANOVA on the experimental data, and the Duncan method was used for multiple comparative analysis.

The comprehensive absorption and purification ability of indoor plants to benzene pollution is calculated using the membership function method. The formula^40^ is as follows:

4 $${U}_{(\text{Xi)}}=\frac{{X}_{i}-{\text{X}}_{\text{min}}}{{X}_{\text{max}}-{\text{X}}_{\text{min}}}$$

In the formula, U_(Xi)_ corresponds to the value of the function, and U_(Xi)_ is ∈ [0, 1]; X_i_ is the measured value of the indicator; X_min_ and X_max_ are the minimum and maximum measured values of the indicators.

### Statement

All plant experiments were conducted in accordance with institutional, national and international guidelines and regulation. All plant collections have been licensed by the nation.

## Results

### Changes in the benzene purification rate of indoor plants under benzene stress

**Figure 1:**
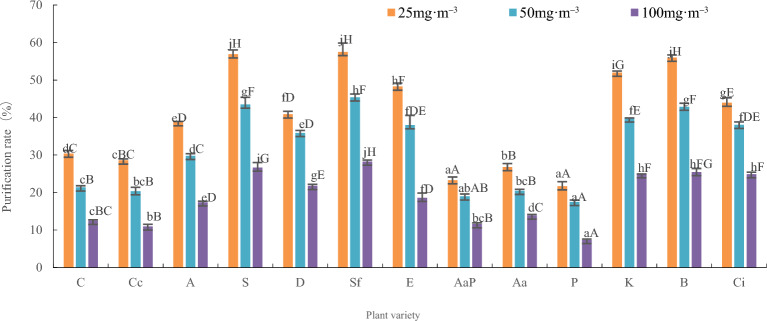
Purification rate of 13 indoor plants at different benzene pollution. C—*Chlorophytum comosum*, Cc— *Chlorophytum capense* var. *variegatum*, A—*Aloe arborescens*, S—*Sansevieria trifasciata* var. *laurentii*, D—*Dieffenbachia picta*, Sf—*Spathiphyllum floribundum*, E—*Epipremnum aureum*, AaP— *Anthurium andraeanum ‘Pink champin’*, Aa—*Anthurium andraeanum*, P— *Podocarpus nagi*, K—*Kalanchoe blossfeldiana*, B—*Begonia xaelatior*, Ci—*Calathea insignis*, a-z means significant difference at level P < 0.05, A-Z means significant difference at level P < 0.01.

The analysis of variance for the plant purification rate is shown in Table 1 and Fig. 1. The thirteen indoor plants showed abilities to absorb and purify benzene at three different concentrations. The influence of plant species, benzene concentration, and the interaction between the two on the purification rate was extremely significant, and the difference in the purification rate was significant (F value): benzene concentration > plant species > plant species × benzene concentration. The influence of benzene concentration on the plant purification rate was more significant than other factors (F concentration > F).

**Table 1:** Two-way ANOVA of benzene purification rate in plants.

Source	df	SS	MS	F	Fα	Comment
Plant	12	1.107	0.092	2708.577**	*F*_0.01_ (12, 78) = 2.42	
Concentration	2	0.925	0.463	13,585.112**	*F*_0.01_ (2, 78) = 4.88	R-Sq = 99.9%
Plant*Concentration	24	0.089	0.004	108.990**	*F*_0.01_ (24, 78) = 2.03	R-Sq (Adjust) = 99.8%
Error	78	0.003	0.000			
Total correction	116	2.123				

The values in the figure represent the correlation coefficient, *represents significantly correlated (P < 0.05), and **represents extremely significantly correlated (P < 0.01).

Under the 25 mg m^–3^ benzene treatment, there were no significant differences in the purification rate among *Podocarpus nagi* and *Anthurium andraeanum 'Pink champin'*, and Begonia xaelatior, *Sansevieria trifasciata* var. *laurentii*, and *Spathiphyllum floribundum*. However, there were significant differences between *Anthurium andraeanum* and *Chlorophytum capense* var. *variegatum*; *Chlorophytum capense* var. *variegatum* and *Chlorophytum comosum*; and *Aloe arborescens* and *Dieffenbachia picta*. There were extremely significant differences among other plant species. The plants showed the following order for the purification rate: *Spathiphyllum floribundum* (57.5%), *Sansevieria trifasciata* var. *laurentii* (56.9%), *Begonia xaelatior* (56.0%), *Kalanchoe blossfeldiana* (52.0%), *Epipremnum aureum* (48.3%), *Calathea insignis* (44.0%), *Dieffenbachia picta* (40.9%), *Aloe arborescens* (38.8%), *Chlorophytum comosum* (30.4%), *Chlorophytum capense* var. *variegatum* (28.6%), *Anthurium andraeanum* (26.8%), *Anthurium andraeanum 'Pink champin'* (23.3%), and *Podocarpus nagi* (21.7%).

At a concentration of 50 mg·m^–3^ benzene, there were no significant differences in the purification rate between *Podocarpus nagi* and *Anthurium andraeanum 'Pink champin'*; *Anthurium andraeanum 'Pink champin'* and *Chlorophytum capense* var. *variegatum* and *Anthurium andraeanum*; *Chlorophytum capense* var. *variegatum* and *Anthurium andraeanum* and *Chlorophytum comosum*; *Epipremnum aureum* and *Calathea insignis* and *Kalanchoe blossfeldiana*; and *Begonia xaelatior* and *Sansevieria trifasciata* var. *laurentii*. However, there were significant differences between *Anthurium andraeanum 'Pink champin'* and *Chlorophytum comosum* and between *Dieffenbachia picta* and *Epipremnum aureum* and *Calathea insignis*. There were extremely significant differences among other plant species. The order of purification rate was as follows: *Spathiphyllum floribundum* (45.4%), *Sansevieria trifasciata* var. *laurentii* (43.5%), *Begonia xaelatior* (42.9%), *Kalanchoe blossfeldiana* (39.8%), *Calathea insignis* (38.1%), *Epipremnum aureum* (38.0%), *Dieffenbachia picta* (35.9%), *Aloe arborescens* (29.8%), *Chlorophytum comosum* (21.4%), *Anthurium andraeanum* (20.5%), *Chlorophytum capense* var. *variegatum* (20.4%), *Anthurium andraeanum 'Pink champin'* (19.0%), and *Podocarpus nagi* (17.5%).

At a concentration of 100 mg·m^-3^ benzene, there were no significant differences in the purification rate between *Chlorophytum capense* var. *variegatum* and *Anthurium andraeanum 'Pink champin'*; *Anthurium andraeanum 'Pink champin'* and *Chlorophytum comosum*; and *Kalanchoe blossfeldiana* and *Calathea insignis* and Begonia xaelatior. However, there were significant differences between *Chlorophytum capense* var. *variegatum* and *Chlorophytum comosum*; *Chlorophytum comosum* and *Anthurium andraeanum*; *Aloe arborescens* and *Epipremnum aureum*; and *Begonia xaelatior* and *Sansevieria trifasciata* var. *laurentii*. There were extremely significant differences among other plant species. The purification rate was in the order *Spathiphyllum floribundum* (28.3%), *Sansevieria trifasciata* var. *laurentii* (26.7%), *Begonia xaelatior* (25.5%), *Kalanchoe blossfeldiana* (24.9%), *Calathea insignis* (24.9%), *Dieffenbachia picta* (21.8%), *Epipremnum aureum* (18.6%), *Aloe arborescens* (17.4%), *Anthurium andraeanum* (13.9%), *Chlorophytum comosum* (12.5%), *Anthurium andraeanum 'Pink champin'* (11.6%), *Chlorophytum capense* var. *variegatum* (11.0%), and *Podocarpus nagi* (7.4%).

The average purification rate of the 13 types of plants under the three benzene levels was ranked from high to low: *Spathiphyllum floribundum* (43.7%), *Sansevieria trifasciata* var. *laurentii* (42.4%), *Begonia xaelatior* (41.5%), *Kalanchoe blossfeldiana* (38.9%), *Calathea insignis* (35.7%), *Epipremnum aureum* (35.0%), *Dieffenbachia picta* (32.9%), *Aloe arborescens* (38.7%), *Chlorophytum comosum* (31.4%), *Anthurium andraeanum* (20.4%), *Chlorophytum capense* var. *variegatum* (20.0%), *Anthurium andraeanum 'Pink champin'* (18.0%), and *Podocarpus nagi* (15.5%). The variation range of the purification rates for the 13 types of plants was 21.7–57.5%, 17.5–45.4%, and 7.4–28.3% at the three benzene levels, respectively. The average purification rates were 40.4%, 31.7%, and 18.8%, respectively. The purification rate showed a decreasing trend with increasing benzene concentration, and the decrease in amplitude increased.

Among the plants, under benzene concentrations of 25 mg m^-3^, 50 mg m^-3^, and 100 mg m^-3^, the highest average purification rate was observed in *Spathiphyllum floribundum*, followed by *Sansevieria trifasciata* var. *laurentii*, and the lowest was seen in *Podocarpus nagi*.

### Changes in the purification capacity per unit leaf area of indoor plants under benzene stress

**Figure 2:**
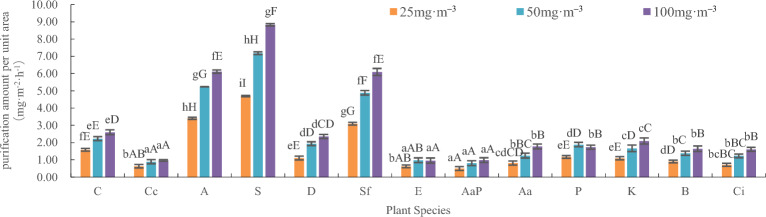
Leaf purification capacity per unit area of 13 indoor plants. C—*Chlorophytum comosum*, Cc— *Chlorophytum capense* var. *variegatum*, A—*Aloe arborescens*, S—*Sansevieria trifasciata* var. *laurentii*, D—*Dieffenbachia picta*, Sf—*Spathiphyllum floribundum*, E—*Epipremnum aureum*, AaP— *Anthurium andraeanum ‘Pink champin’*, Aa—*Anthurium andraeanum*, P— *Podocarpus nagi*, K—*Kalanchoe blossfeldiana*, B—*Begonia xaelatior*, Ci—*Calathea insignis*, a-z means significant difference at level P < 0.05, A-Z means significant difference at level P < 0.01.

The analysis of variance for purification capacity per unit leaf area is shown in Table 2 and Fig. 2. The thirteen indoor plants had the ability to absorb and purify benzene at three different concentrations. The influence of plant species, benzene concentration, and the interaction between the two on the purification rate was extremely significant, and the purification capacity per unit leaf area was significant (F value): plant species > benzene concentration > plant species × benzene concentration. However, the effect of plant species on purification capacity per unit leaf area was more significant (F plant species > F).

**Table 2:** Two-way ANOVA of plant purification of benzene per unit leaf area.

Source	df	SS	MS	F	Fα	Comment
Plant	12	390.415	32.535	194.169**	*F*_0.01_ (12,78) = 2.42	
Concentration	2	37.088	18.544	110.672**	*F*_0.01_ (2,78) = 4.88	R-Sq = 97.2%
Plant*Concentration	24	26.264	1.094	6.531**	*F*_0.01_ (24,78) = 2.03	R-Sq (Adjust) = 95.8%
Error	78	13.070	0.168			
Total correction	116	446.836				

The values in the figure represent the correlation coefficient, *represents significantly correlated (P < 0.05), and **represents extremely significantly correlated (P < 0.01).

At a 25 mg m^-3^ benzene concentration, there was no significant difference in the purification capacity per unit leaf area between *Calathea insignis* and *Anthurium andraeanum*; *Anthurium andraeanum* and Begonia xaelatior; *Epipremnum aureum* and *Chlorophytum capense* var. *variegatum* and *Calathea insignis*; and *Kalanchoe blossfeldiana* and *Dieffenbachia picta* and *Podocarpus nagi*. However, there were significant differences among *Anthurium andraeanum 'Pink champin'* and *Epipremnum aureum* and *Chlorophytum capense* var. *variegatum*. There were extremely significant differences among other plant species. The order of purification amount per unit leaf area was as follows: *Sansevieria trifasciata* var. *laurentii* (4.70 mg·m^–2^·h^–1^), *Aloe arborescens* (3.40 mg·m^–2^·h^–1^), *Spathiphyllum floribundum* (3.09 mg·m^–2^·h^–1^), Chlorophytum comosum (1.58 mg·m^–2^·h^–1^), *Podocarpus nagi* (1.17 mg·m^–2^·h^–1^), *Dieffenbachia picta* (1.10 mg·m^–2^·h^–1^), *Kalanchoe blossfeldiana* (1.09 mg·m^–2^·h^–1^), *Begonia xaelatior* (0.90 mg·m^–2^·h^–1^), *Anthurium andraeanum* (0.81 mg·m^–2^·h^–1^), *Calathea insignis* (0.71 mg·m^–2^·h^–1^), *Chlorophytum capense* var. *variegatum* (0.63 mg·m^–2^·h^–1^), *Epipremnum aureum* (0.62 mg·m^–2^·h^–1^), and *Anthurium andraeanum 'Pink champin'* (0.49 mg·m^–2^·h^–1^).

At a concentration of 50 mg·m^-3^ benzene, there was no significant difference in the purification capacity per unit leaf area between *Anthurium andraeanum 'Pink champin'* and *Chlorophytum capense* var. *variegatum* and *Epipremnum aureum*; *Calathea insignis* and *Anthurium andraeanum* and Begonia xaelatior; and *Podocarpus nagi* and *Dieffenbachia picta*. However, there was a significant difference between *Epipremnum aureum* and *Calathea insignis* and *Anthurium andraeanum* and between *Kalanchoe blossfeldiana* and *Podocarpus nagi* and *Dieffenbachia picta*. There were extremely significant differences among other plant species. The order of purification amount per unit leaf area was as follows: *Sansevieria trifasciata* var. *laurentii* (7.18 mg·m^–2^·h^–1^), *Aloe arborescens* (5.24 mg·m^–2^·h^–1^), *Spathiphyllum floribundum* (4.89 mg·m^–2^·h^–1^), *Chlorophytum comosum* (2.23 mg·m^–2^·h^–1^), *Dieffenbachia picta* (1.93 mg·m^–2^·h^–1^), *Podocarpus nagi* (1.88 mg·m^–2^·h^–1^), *Kalanchoe blossfeldiana* (1.67 mg·m^–2^·h^–1^), *Begonia xaelatior* (1.38 mg·m^–2^·h^–1^), *Anthurium andraeanum* (1.25 mg·m^–2^·h^–1^), *Calathea insignis* (1.23 mg·m^–2^·h^–1^) *Epipremnum aureum* (0.97 mg·m^–2^·h^–1^), *Chlorophytum capense* var. *variegatum* (0.89 mg·m^–2^·h^–1^), and *Anthurium andraeanum 'Pink champin'* (0.80 mg·m^–2^·h^–1^).

Under the 100 mg·m^–3^ benzene treatment, there was no significant difference in the purification capacity per unit leaf area between *Epipremnum aureum* and *Chlorophytum capense* var. *variegatum*; *Anthurium andraeanum 'Pink champin'*, *Calathea insignis* and Begonia xaelatior; and *Podocarpus nagi* and *Anthurium andraeanum*, *Spathiphyllum floribundum* and *Aloe arborescens*. However, there were significant differences between *Kalanchoe blossfeldiana* and *Dieffenbachia picta* and between *Dieffenbachia picta* and *Chlorophytum comosum*. There were extremely significant differences among other plant species. The order of purification amount per unit leaf area was as follows: *Sansevieria trifasciata* var. *laurentii* (8.83 mg·m^–2^·h^–1^), *Aloe arborescens* (6.11 mg·m^–2^·h^–1^), *Spathiphyllum floribundum* (6.09 mg·m^–2^·h^–1^), *Chlorophytum comosum* (2.60 mg·m^–2^·h^–1^), *Dieffenbachia picta* (2.35 mg·m^–2^·h^–1^), *Kalanchoe blossfeldiana* (2.09 mg·m^–2^·h^–1^), *Anthurium andraeanum* (1.77 mg·m^–2^·h^–1^), *Podocarpus nagi* (1.73 mg·m^–2^·h^–1^), *Begonia xaelatior* (1.64 mg·m^–2^·h^–1^) *Calathea insignis* (1.61 mg·m^–2^·h^–1^), *Anthurium andraeanum 'Pink champin'* (0.98 mg·m^–2^·h^–1^), *Chlorophytum capense* var. *variegatum* (0.97 mg·m^–2^·h^–1^), and *Epipremnum aureum* (0.95 mg·m^–2^·h^–1^).

The average purification capacity per unit leaf area of the 13 plants under the three levels of benzene stress was as follows: *Sansevieria trifasciata* var. *laurentii* (6.90 mg·m^–2^·h^–1^), *Aloe arborescens* (4.92 mg·m^–2^·h^–1^), *Spathiphyllum floribundum* (4.69 mg·m^–2^·h^–1^) *Chlorophytum comosum* (2.13 mg·m^–2^·h^–1^), *Dieffenbachia picta* (1.80 mg·m^–2^·h^–1^), *Kalanchoe blossfeldiana* (1.62 mg·m^–2^·h^–1^), *Podocarpus nagi* (1.59 mg·m^–2^·h^–1^), *Begonia xaelatior* (1.31 mg·m^–2^·h^–1^), *Anthurium andraeanum* (1.28 mg·m^–2^·h^–1^), *Calathea insignis* (1.18 mg·m^–2^·h^–1^), *Epipremnum aureum* (0.85 mg·m^–2^·h^–1^), *Chlorophytum capense* var. *variegatum* (0.83 mg·m^–2^·h^–1^), and *Anthurium andraeanum 'Pink champin'* (0.76 mg·m^–2^·h^–1^). The range of purification amount per unit leaf area of the 13 plants was 4.7 mg·m^–2^·h^–1^ ~ 1.17 mg·m^–2^·h^–1^, the change in the range of the purification amount per unit leaf area of the 13 plants was 7.18 mg·m^–2^·h^–1^ ~ 1.88 mg·m^–2^·h^–1^, and the change in the range of the purification amount per unit leaf area of the 13 plants was 8.83 mg·m^–2^·h^–1^ ~ 1.93 mg·m^–2^·h^–1^. The average purification capacities were 1.56 mg·m^–2^·h^–1^, 2.42 mg·m^–2^·h^–1^, and 2.90 mg·m^–2^·h^–1^ at the three concentrations, respectively. With increasing benzene concentration, the purification amount per unit leaf area of the plants showed an upwards trend, and the increase rate decreased.

Among the plants, under benzene concentrations of 25 mg m^–3^, 50 mg m^–3^, and 100 mg m^–3^, the highest average purification amount per unit leaf area was observed in *Sansevieria trifasciata* var. *laurentii*, followed by *Aloe arborescens*, and the lowest was seen in *Anthurium andraeanum 'Pink champin'*.

### Comprehensive evaluation and analysis of the absorption and purification capacity of indoor plants under benzene stress

Comprehensive evaluation was performed for the purification rates and the per unit leaf area purification amount of 13 indoor plants under different concentrations of benzene through membership functions (Table 3). Based on comprehensive evaluation results, the absorption and purification capacity of the 13 plants are ranked as follows: *Sansevieria trifasciata* var. *laurentii*, *Spathiphyllum floribundum*, *Aloe arborescens*, Begonia xaelatior, *Kalanchoe blossfeldiana*, *Calathea insignis*, *Dieffenbachia picta*, *Epipremnum aureum*, *Chlorophytum comosum*, *Anthurium andraeanum*, *Chlorophytum capense* var. *variegatum*, *Podocarpus nagi*, and *Anthurium andraeanum 'Pink champin'*. The absorption and purification ability of *Sansevieria trifasciata* var. *laurentii Spathiphyllum floribundum* and *Aloe arborescens* were the strongest, and those of *Podocarpus nagi* and *Anthurium andraeanum 'Pink champin'* were the weakest.

**Table 3:** Comprehensive assessment of the decontamination capacity of experimental plants against benzene pollution.

Plant variety	Purification rate (%)	Purification amount per unit leaf area (mg·m^–2^·h^–1^)	Comprehensive evaluation value	Ranking of absorption and purification capacity
*Sansevieria trifasciata var. laurentii*	0.947	1	0.97	1
*Spathiphyllum floribundum*	1	0.64	0.82	2
*Aloe arborescens*	0.466	0.68	0.57	3
*Begonia xaelatior*	0.911	0.09	0.50	4
*Kalanchoe blossfeldiana*	0.828	0.14	0.48	5
*Calathea insignis*	0..733	0.07	0.40	6
*Dieffenbachia picta*	0.629	0.02	0.40	7
*Epipremnum aureum*	0.672	0.23	0.35	8
*Chlorophytum comosum*	0.208	0.08	0.22	9
*Anthurium andraeanum*	0.188	0.02	0.14	10
*Chlorophytum capense var. variegatum*	0.157	0.14	0.09	11
*Podocarpus nagi*	0	0	0.07	12
*Anthurium andraeanum ‘Pink champin’*	0.101		0.05	13

## Discussion

### Effect of plant species on benzene purification ability

This study have shown that different plants differ in their ability to absorb and purify benzene^35^. Some studies have found that succulent leaf plants, such as those in the Agave family and Lily family^41^, and leathery leaf plants in the Amanita family^42^, have a strong ability to purify benzene pollution. All these studies are similar to the results of the present study. From the perspective of purification rates, the purification amount per unit leaf area, and comprehensive purification ability under three concentrations of benzene, *Sansevieria trifasciata* var. *laurentii* represented by Agavaceae, *Aloe arborescens* represented by Liliaceae, *Begonia xaelatior* represented by Begonia, *Kalanchoe blossfeldiana* represented by Crassulaceae, and *Spathiphyllum floribundum* represented by Araceae, showed the strongest purification ability. In contrast, *Chlorophytum capense* var. *variegatum* and *Chlorophytum comosum* of Liliaceae had weak purification capacity, which may be due to differences in leaf texture, leaf area, leaf stomata and density, and the arrangement of mesophyll cells in different plants^43,44^. While the ability of plants to absorb air pollutants is less related to stomatal density and cuticle thickness^43^, but more related to the composition and content of keratin and waxes^33,45, 46^; the epidermis of succulent polypods is rich in keratin and waxes, and palmitic acid is the main component of the wax layer of polypod plants, which has a strong capacity for benzene absorption^47^. It has also been found that the change rate of plant physiological and biochemical indices under high benzene concentration is significant and has relatively high benzene purification efficiency^12^, which is also similar to the study results. In addition, Lu, et al.^18^ found that under stress from low-concentration benzene pollution, the purification capacity of *Sansevieria trifasciata* var. *laurentii* and *Spathiphyllum floribundum* were the strongest among nine indoor plants, and the purification capacity of *Chlorophytum comosum* plants was weak, which is consistent with the conclusions of this study. Therefore, both succulent leafy plants and Araceae leathery leafy plants have strong purification capabilities for benzene pollution.

### The effect of benzene concentration on the absorption and purification ability of plants

Some studies have shown that there was a negative correlation between benzene concentration and plant purification rate, meaning that the plant purification rate decreased with increasing benzene concentration and there was a positive correlation of benzene concentration with the purification amount per unit leaf area; that is, with increasing benzene concentration, the purification amount per unit leaf area of plants increased^18^. This is consistent with the findings of this study. In this study, compared to a concentration of 25 mg·m^–3^ benzene, the decrease in the plant purification rate was only 21.5% at a concentration of 50 mg·m^–3^ benzene. The plant purification rate decreased by 40.7% at a concentration of 100 mg·m^–3^ benzene compared to 50 mg·m^-3^ benzene pollution. The plant purification rate decreased by 53.5% at a concentration of 100 mg·m^–3^ benzene compared to a concentration of 25 mg·m^–3^ benzene. The purification amount per unit leaf area of plants increased by 85.9% at a concentration of 100 mg·m^–3^ benzene compared to 25 mg·m^-3^ benzene. Compared to a concentration of 25 mg·m^–3^ benzene, the purification amount per unit leaf area of plants increased by 55.1% at a concentration of 50 mg·m^–3^ benzene. Compared to a concentration of 50 mg·m^–3^ benzene, the purification rate per unit leaf area of plants increased by only 19.8% at a concentration of 100 mg·m^–3^ benzene, which is similar to the results of low-concentration benzene pollution reported by Lu et al.^18^. The purification rate of plants under 100 mg·m^–3^ benzene stress decreased to varying degrees compared to 50 mg·m^–3^ and 25 mg·m^–3^ benzene stress, and the decrease in amplitude increased. The purification amount per unit leaf area of plants under 100 mg·m^–3^ benzene stress increased to varying degrees compared to the purification amount per unit leaf area under 25 mg·m^–3^ and 50 mg·m^–3^ benzene stress, and the increase rate decreased. It is speculated that 50 mg·m^–3^ benzene pollution is close to the saturation concentration for plant benzene absorption, and 100 mg·m^–3^ benzene pollution is close to the critical concentration that plants can tolerate.

### Comparison of analysis methods for the benzene purification ability of plants

The ability of plants to purify benzene pollution is represented and evaluated by different indicators, each of which displays purification ability to varying degrees, and the impact of different indicators on plant purification ability is also different. This study revealed that among *Spathiphyllum floribundum*, *Sansevieria trifasciata* var. *laurentii*, Begonia xaelatior, *Kalanchoe blossfeldiana* and *Calathea insignis*, which showed high purification rates, the purification capacities per unit leaf area of *Kalanchoe blossfeldiana*, *Begonia xaelatior* and *Calathea insignis* were low. Among the high purification capacity per unit leaf area of *Sansevieria trifasciata* var. *laurentii*, *Aloe arborescens*, *Spathiphyllum floribundum*, *Chlorophytum comosum*, and Dieffenbachia picta, the purification rates of Aloe arborescens and Chlorophytum comosum arborescens were low, which may be related to plant size. The size and quantity of leaves are related^1,30^. Most scholars only use a single indicator consisting of purification rate or purification amount per unit leaf area to evaluate the purification ability of plants for benzene pollution^47,48^, ignoring the impact of factors such as the size of plant leaf area and biomass on plant purification ability. Therefore, to avoid the one-sidedness, limitations, and instability caused by the use of single indicators, the membership function method was used in this study to comprehensively evaluate and analyse the purification rate and purification amount per unit leaf area, and the obtained results are more realistic and objective. In addition, it has been suggested that extracellular enzymes secreted by plant inter-root microorganisms can have some degrading effect on pollution^49^. The soil of potted plants and pot parts were wrapped with plastic film during the experiment to eliminate the potential confounding effects of soil microorganisms and pots on benzene purification. This approach ensured that the plant microorganisms and soil effects were effectively controlled, allowing for a more accurate assessment of the specific impact of plants on the benzene.

## Conclusions

This study revealed the absorption and purification ability of indoor plants to benzene pollution by analysing the purification rates and purification amount per unit leaf area of 13 indoor plants under different high concentrations of benzene stress. The results showed that there were significant differences in the absorption and purification capacity of different plant species for indoor benzene pollution. The effects of plant type, benzene concentration, and their interaction on the purification rate and purification amount per unit leaf area of 13 indoor plants were extremely significant. Among the factors, benzene concentration had a more significant impact on the purification rate, while plant species had a more significant impact on the purification amount per unit leaf area. As the concentration of benzene increased, the purification rate of plants gradually decreased, and the decreasing trend increased. The purification amount per unit leaf area of plants gradually increased, and the increasing trend decreased. Preliminary screening identified several indoor plants with strong absorption and purification capabilities for benzene pollution, including *Sansevieria trifasciata* var. *laurentii*, *Spathiphyllum floribundum*, *Aloe arborescens*, Begonia xaelatior, and *Kalanchoe blossfeldiana*. Among them, *Sansevieria trifasciata* var. *laurentii* is considered the preferred plant for purifying and remediating indoor benzene pollution. The results can provide a theoretical basis and reference for the selection of plants with strong absorption and purification capabilities for indoor benzene pollution.

## Data Availability

The datasets used and/or analysed during the current study available from the corresponding author on reasonable request.
